# Silicon‐mediated multiple interactions: Simultaneous induction of rice defense and inhibition of larval performance and insecticide tolerance of *Chilo suppressalis* by sodium silicate

**DOI:** 10.1002/ece3.6235

**Published:** 2020-04-12

**Authors:** Jie Wang, Rongrong Xue, Xueyang Ju, Hui Yan, Zhou Gao, Mohammed Esmail Abdalla Elzaki, Lin Hu, Rensen Zeng, Yuanyuan Song

**Affiliations:** ^1^ Key Laboratory of Ministry of Education for Genetics, Breeding and Multiple Utilization of Crops College of Agriculture Fujian Agriculture and Forestry University Fuzhou China; ^2^ State Key Laboratory of Ecological Pest Control for Fujian and Taiwan Crops Fujian Agriculture and Forestry University Fuzhou China

**Keywords:** *Chilo suppressalis*, chlorpyrifos, detoxifying enzyme, insecticide resistance, *Oryza sativa*, plant defense, silicon

## Abstract

The rice striped stem borer (SSB, *Chilo suppressalis*) is one of the most destructive pests of rice plants. Si‐mediated rice defense against various pests has been widely reported, and sodium silicate (SS) has been used as an effective source of silicon for application to plants. However, there is quite limited information about the direct effects of Si application on herbivorous insects. SSB larval performance and their insecticide tolerance were examined after they had been reared either on rice plants cultivated in nutrient solution containing 0.5 and 2.0 mM SS or on artificial diets with 0.1% and 0.5% SS. SS amendment in either rice culture medium or artificial diets significantly suppressed the enzymatic activities of acetylcholinesterase, glutathione S‐transferases, and levels of cytochrome P450 protein in the midgut of *C. suppressalis* larvae. Larvae fed on diets containing SS showed lower insecticide tolerance. Additionally, RNA‐seq analysis showed that SS‐mediated larval insecticide tolerance was closely associated with fatty acid biosynthesis and pyruvate metabolism pathways. Our results suggest that Si not only enhances plant resistance against insect herbivore, but also impairs the insect's capacity to detoxify the insecticides. This should be considered as another important aspect in Si‐mediated plant–insect interaction and may provide a novel approach of pest management.

## Graphical abstract

Sodium silicate (SS) amendments in rice plants significantly suppressed the activities of insecticide detoxification‐related enzymes acetylcholinesterase, glutathione S‐transferases, and cytochrome P450 in *Chilo suppressalis* larvae. Therefore, larvae fed on SS‐amended plants showed lower insecticide tolerance. Double‐check the phenomena above by using artificial diets, we found sodium silicate had directly adverse effects on the performance of *C. suppressalis* larvae to further insecticide treatments.

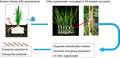

## 1 INTRODUCTION

The rice striped stem borer (SSB), *Chilo suppressalis* (Walker) (Lepidoptera: Crambidae), is one of the most destructive pests of rice (*Oryza sativa* L.) in most of rice‐growing regions (Cheng, Chang, & Dai, 2010; Djamin & Pathak, 1967). The larvae feed on the epidermis in the inner side of leaf sheath and bore into rice stalk and may cause deadhearts and whiteheads during the vegetative and reproductive stages, respectively (Pathak, 1968). Control of this pest relies heavily on insecticides, especially organophosphates, methyl carbamates, and phenylpyrazole insecticides (Cheng et al., 2010; Jiang et al., 2009; Li et al., 2017; Zibaee, Sendi, Ghadamyari, Alinia, & Etebari, 2009), while the efficiency of insecticides on controlling this pest is generally low due to the narrow window of exposure resulting from boring into rice stalk after the larvae reach 2nd instar (Yue et al., 2008; Sheng, Wang, Sheng, Gao, & Xuan, 2003). Additionally, long‐term and intensive applications of insecticides have driven SSB to evolve resistance by enhancing specific enzymes such as carboxylesterase, glutathione S‐transferases, cytochrome P450s, microsomal‐*O*‐demethylase, and acetylcholinesterase (Cao, Shen, & Liu, 2001; Jiang et al., 2009; Li, Huang, Yuan, & Tang, 2007; Qu, Han, Xu, & Yue, 2003). Thus, exploiting plant resistance could be an economically and ecologically efficient approach for integrated pest management.

Silicon (Si) is the second most common element after oxygen in the earth's crust (Epstein, 1994). In addition, Si has been listed as a “beneficial substance” by the International Plant Nutrition Institute (IPNI, 2015), which has been widely reported to improve plant resistance against both abiotic and biotic stresses (Bhat, Nazir, Mahajan, Zargar, & Deshmukh, 2019; Liang, Nikolic, Bélanger, Haijun, & Alin, 2015; Liang, Sun, Zhu, & Christie, 2007; Ma, 2004; Meharg & Meharg, 2015; Wang et al., 2017). In the performance assay, the addition of Si to maize resulted in increased larval mortality of the true armyworm, *Pseudeletia unipuncta* (Haworth) compared with the maize plants without Si (Moise, McNeil, Hartley, & Henry, 2019). Therefore, application of Si is a potential management method to control a wide range of pests including leaf‐chewing (Han, Lei, Wen, & Hou, 2015; Ye et al., 2013), sap‐feeding (Dias et al., 2014; Goussain, Prado, & Moraes, 2005), and stem‐boring insects (Hou & Han, 2010; Kvedaras & Keeping, 2007). However, the results of foliar‐applied Si on plant resistance against biotic stress such as pests sometimes are considered controversial because current evidence suggests that Si needs to be absorbed by plant roots to trigger systemic resistance (Coskun et al., 2018). For enhanced resistance to pests by application of Si to plants, an alternative explanation is that insects could directly consume soluble Si which may have direct effects on insect physiology. However, little information has been drawn on the direct effect of Si on insects and its related mechanisms. Thus, the scenario beyond Si directly mediating plant–insect interactions deserved further investigation.

Sodium silicate (SS) has been used as an effective source of Si (Heckman, 2013). Application of SS to plants has been shown to influence insect performance. For example, Italian ryegrass (*Lolium multiflorum*) fertilized with SS demonstrated a reduction in colonization by stem‐boring larvae of *Oscinella frit* compared to the control plants (Moore, 1984). Application of SS to wheat plants significantly reduced preference, longevity, and production of nymphs of *Schizaphis graminum* (Basagli et al., 2003; Moraes et al., 2004). Similarly, both foliar and soil application of another type of soluble Si (silicic acid) enhanced rice resistance against fall armyworm (Nascimento, Assis, Moraes, & Souza, 2018).

There is growing and compelling evidence that adaptation to toxic host plants has been a factor in the evolution of insecticide resistance in some herbivore species (Alyokhin & Chen, 2017; Ryan & Byrne, 1988). For example, the susceptibility of *Spodoptera littoralis* to pesticides differs with host plants by impacting detoxification enzyme levels (Abd El‐Rahman, Salem, Yacoub, & Naguib, 2019). Because it is possible for SSB larvae to directly consume Si, we hypothesized that SS exposures may also directly impact the pest's ability to detoxify insecticides. Insect herbivores rely heavily on their detoxification enzymes typically including the glutathione S‐transferases (GSTs), cytochrome P450 monooxygenases (P450s), and carboxylesterases to overcome the toxicity of allelochemicals in host plants and insecticides (Després, David, & Gallet, 2007; Terriere, 1984).

This work aimed to characterize the role of SS in SSB larval performance, resistance‐related enzymes (AChE, GST, and CYP450), differential gene expression, and insecticide tolerance. Results of the present study may expand the current understanding of the beneficial aspects of Si to be used as an environment‐friendly agent for pest management purpose.

## 2 MATERIALS AND METHODS

### 2.1 Insects

The *C. suppressalis* population was initially collected in 2016 from rice paddy fields on the campus of Fujian Agriculture and Forestry University (Fuzhou, China) and maintained under laboratory conditions. Larvae were reared on artificial diets at 25 ± 2°C and 70 ± 5% relative humidity with a photoperiod of 16:8 hr (L:D), and artificial diets were prepared according to the protocol of Liu, Li, Han, Peng, and Hou (2012).

### 2.2 Plants

Rice (*O. sativa* L. cv. Nipponbare) seeds were surface‐sterilized with 2% sodium hypochlorite (v/v) for 20 min and then were rinsed with distilled water twice. The seeds were soaked in Milli‐Q water for 2 days and then were placed on moist filter paper for a week in Petri dishes at 25℃. After germination, rice seedlings with uniform size were transferred to the polyethylene pots and hydroponically cultured in a nutrient solution that contains both macronutrients and micronutrients for the growth of rice plants (Yoshida, Forno, Cock, & Gomez, 1976). Plants were grown in a greenhouse with a day:night temperature regime of 32℃ (12 hr): 26℃ (12 hr), 75% relative humidity, and nature daylight. Nutrient solutions were replenished every 3 days, and rice plants were used for all the experiments 20 days after transplanting.

### 2.3 Effect of Si application to rice plants on SSB performance and insecticide tolerance

To examine whether Si supplement induced rice defense against SSB larvae, rice plants were exposed to a nutrient solution containing 2.0 mM sodium silicate (SS, Na_2_SiO_3_∙9H_2_O). For Si negative treatments, 2.0 mM sodium chloride was added to balance sodium levels. A plastic straw (diameter: 3 cm, length: 6 cm) was used to fix larvae on the rice stem with cotton plugging the both ends to prevent insects from crawling away. Newly molted 3rd‐instar SSB larvae (20 ± 5 mg) were selected and allowed to feed on the stem. Boring behavior was also observed within 90 min by recording the time of penetration and start of boring (Hou & Han, 2010). Each treatment had 48 replicates. Percentage of penetration was calculated as the number of larvae successfully penetrating divided by the total number of larvae inoculated. After 3 days, the larvae were removed and weighted. The Si concentration in the rice stem was measured using molybdenum‐blue spectrophotometry method described by Jia, Yang, Zhang, Fang, and Chen (2016). Larvae with similar mass (25 ± 5 mg) were selected and then treated with 2 μl of chlorpyrifos with 98% purity purchased from J&K Scientific Ltd. dissolved in acetone on the larval pronotum. The concentration of chlorpyrifos was 250 μg/ml, which was determined based on the lethal dose (LD_50_). After 48 hr, the larval mortality was recorded. Each treatment had three replicates, and each replicate had 20 individuals.

### 2.4 Direct effects of SS application in artificial diets on SSB growth and tolerance against insecticides

Newly molted 3rd‐instar SSB larvae (20 ± 5 mg) were fed on artificial diets containing sodium silicon (SS) at concentrations of 0.1% and 0.5% (w/w) for 48 hr. For the control treatment, larvae were fed on artificial diets without SS addition. Artificial diets were made based on soybean (*Glycine max*) powder and fresh water bamboo (*Zizania caduciflor*) (Liu et al., 2012). The pH of diets containing sodium silicate was amended with hydrochloric acid to get as similar pH (8.5 ± 0.5) as the control diet. Twenty newly molted 3rd‐instar larvae were used for each treatment, and three independent replicates were conducted for all the treatments. Larval mass was measured at both the beginning and the end of the bioassay for calculating mass gain. After feeding on artificial diets with SS for 48 hr, larvae with similar mass were selected and used for measuring the mortality against chlorpyrifos as described above.

### 2.5 Toxicity of mixture of sodium silicate and insecticide to SSB larvae

The mixture of three different doses of chlorpyrifos ranging from 0.015% to 0.025% (w/w) and two different doses of SS at 0.1% and 0.5% (w/w) were applied into artificial diets synchronously. The newly molted 3rd‐instar larvae were reared on diets supplemented with the mixture of SS and chlorpyrifos for 48 hr, and then, larval mortalities were recorded.

### 2.6 Enzyme extraction and assays

The midguts of larvae fed on both rice plants and artificial diets with or without SS amendment were dissected, and midguts from three larvae were pooled as one replicate for the enzyme assay. To measure the activities of the detoxification‐related enzymes, the dissected midguts were homogenized in 0.1 M PBS (pH 7.6). The homogenate was centrifuged at 4℃, 14,000 *g* for 20 min. The supernatant of each sample was used immediately for the measurement of enzyme activities. Detection kits from Nanjing Jiancheng Bioengineering were used to measure the activities of AChE and GST (Han, Wen, & Hou, 2016). The protein concentration was determined using Bradford assay (Bradford, 1976). For CYP450 enzyme measurement, ELISA Kit (Shanghai Enzyme‐linked Biotechnology Co., Ltd) was used to determine CYP450 levels in insect midguts by using purified insect CYP450 antibody according to manufacturer's instructions, and the concentration of CYP450 in the samples was then determined by comparing the OD value of the samples to the standard curve.

### 2.7 Total RNA isolation

Total RNA was extracted from the midguts of SSB larvae fed on artificial diets with or without SS amendment by use of Total RNA Extraction Kit (Promega Corporation) according to the manufacturer's protocol. Quantity of RNA was confirmed with Nanodrop (Bio‐Rad), and quality of RNA was monitored by electrophoresis gel analysis. Small aliquots of the isolated RNA were stored in −80°C for quantitative real‐time PCR (qRT‐PCR), and the remaining of RNA from the three replicates was used for RNA sequencing.

### 2.8 Library preparation and sequencing

Sequencing libraries were generated using NEBNext® Ultra™ RNA Library Prep Kit for Illumina^®^ (NEB) according to manufacturer's instructions. Purification of mRNA was conducted using NEBNext Poly (A) mRNA Magnetic Isolation Module (NEB). RNA transcript was sequenced on an Illumina Hiseq 2500 platform at Novogene Bioinformatics Institute, and paired‐end reads were generated.

### 2.9 Transcriptome sequencing analysis

Raw data in FASTQ format were processed through in‐house Perl scripts to remove reads containing adapters, reads containing ploy‐N, and low‐quality reads, and the sequencing quality was assessed by measuring Q20, Q30, and GC‐content of the cleaned data. A de novo transcriptome was assembled using the short‐read assembly program Trinity v.2.1.1 (Grabherr et al., 2011). Reads were mapped to unigenes by the Bowtie software (Langmead, 2010), and then, the number of reads mapping to each unigene was converted as FPKM (Fragments Per Kilobase per Million mapped reads) by using RSEM software (Li & Dewey, 2011). To annotate the obtained unigenes, the NCBI nr (nonredundant) and the Swiss‐Prot databases with an E‐value cutoff of 10^−5^ was searched. Functional annotation by KEGG with an E‐value cutoff of 10^–10^ was conducted by searching against the KEGG databases. The read counts were normalized using the edgeR Bioconductor (Robinson, McCarthy, & Smyth, 2009) with the TMM method (Strorey, 2003), and the DESeq R package provided statistical routines for determining differential expression between the control and SS‐treated samples using a model based on the negative binomial distribution. We used “fold changes ≥ 2 and *q* < 0.005” as the threshold to assess DEGs between the SS treatment and control groups. The *p*‐values in multiple tests were adjusted as *q*‐values using the Benjamini and Hochberg's approach for controlling the false discovery rate (Dillies et al., 2013).

### 2.10 Quantitative real‐time PCR analysis

To validate the DEG analysis results, qRT‐PCR experiments were performed in a 10 µl reaction volume consisting of 5 µl of 2× SYBR GoTaq qPCR Master Mix (Promega Corporation), 0.4 µl of each gene‐specific primer (10 µM), 1 µl cDNA equivalent to 50 ng total RNA and sterilized water to reach the final volume. PCR conditions were set as one cycle of 95℃ for 10 min; 40 cycles of 95℃ for 15 s, 55℃ for 30 s, and 72℃ for 30 s. The reference gene elongation factor 1 alpha (*EF‐1α*) was used as the internal control. A dissociation curve analysis program was performed to check the homogeneity of the PCR product. The relative mRNA levels were normalized against *EF‐1α* using the 2^−ΔΔCt^ method (Livak & Schmittgen, 2001). All the primers used for qRT‐PCR were listed on Table 1.

**TABLE 1:** The primers used for qRT‐PCR

	Gene name	Sequence (5′−3′)	GenBank ID
1	*FASN*‐F	AGCAGCGTCGTCTCAGGTAGC	XP_011555838.1
	*FASN*‐R	TGGTACAGTGGCGGCATCCTC	
2	*ALDH*‐F	CCTTCAAGCCAGACACAGAGCAG	AK403889.1
	*ALDH*‐R	TCATCCTTGACATCGGCGAACAC	
3	*HOGH‐F*	TGGGTATTCGTGACTGATAAGAACA	XM_004932486.3
	*HOGH‐R*	GCCAGACGGCAACGAATTTA	
4	*DLDH1‐F*	TTCACGGACCAATGTTGGCTCAC	NM_001043589.1
	*DLDH1‐R*	ACCGACGGAATTGCATCGTAGTTG	
5	*EST*‐F	GTGAGATGGTCCCAAAGTT	ADF43483.1
	*EST*‐R	GGACTCCTTCTTGGCTCT	
6	*CYP6AE60*‐F	TCCGCATTTAAAGCCTTCCAC	KF701137.1
	*CYP6AE60*‐R	ACGGGCTCTGTCCCATAGTA	
7	*EF*‐1α‐F	TGAACCCCCATACAGCGAATCC	XM_022965581.1
	*EF*‐1α‐R	TCTCCGTGCCAACCAGAAATAGG	

### 2.11 Statistical analysis

Gut enzyme assays and Gene expression in the guts of SSB larvae were compared using Student's *t* test. Larval mass gain and larval mortality to insecticides were determined with one‐way ANOVA followed by Fisher's least significant difference (LSD) test. Minitab 17 (Minitab Inc., State College) was used for all analyses. A *p*‐value ≤ .05 for difference between means was considered as significant, and all data were presented as the mean ± *SEM*. Graphs were generated using Graphpad Prism 5 (GraphPad Software Inc.).

## 3 RESULTS

### 3.1 Larval performance and insecticide sensitivity of SSB fed on rice plants with sodium silicate amendment

**FIGURE 1:**
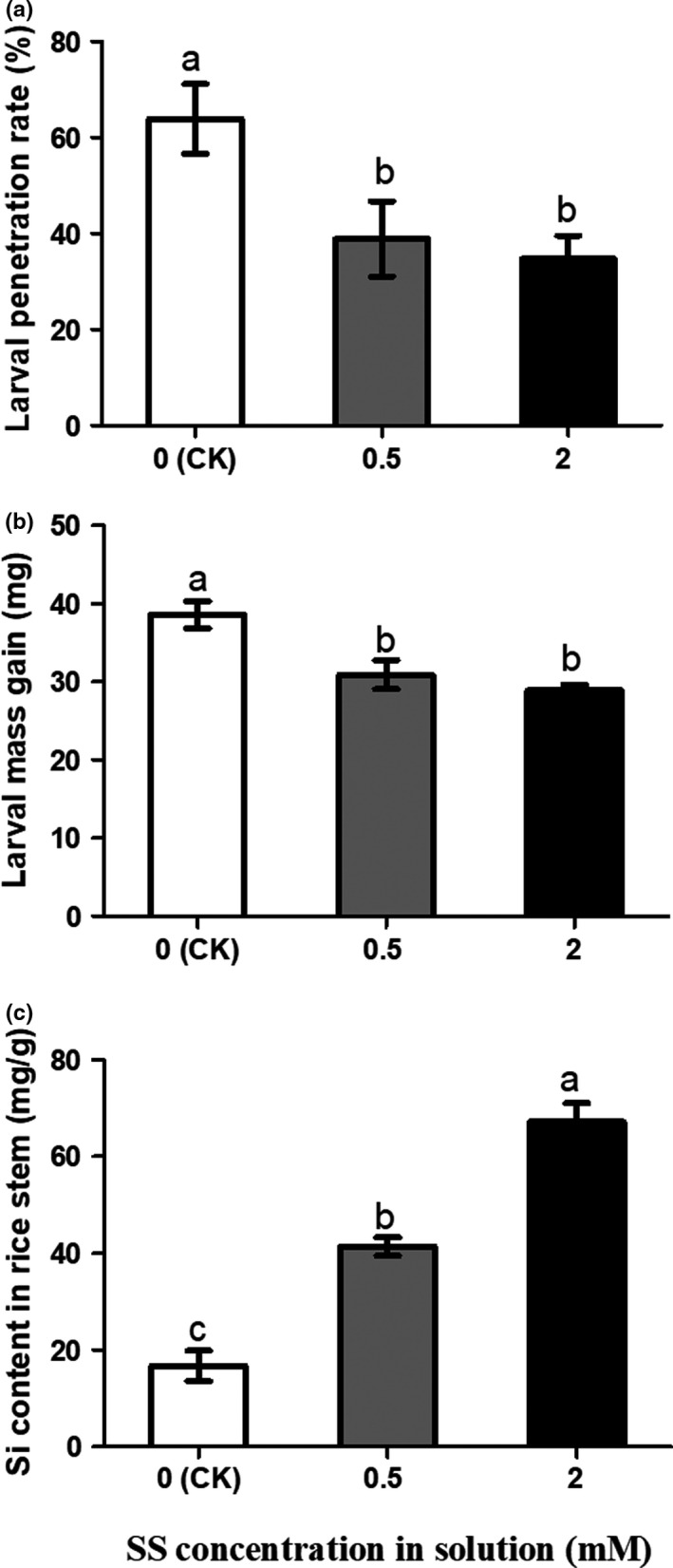
Sodium silicate (SS) induced rice defense against *Chilo suppressalis* larvae along with enhancement of Si content in the stem of rice plant. (a) Larval penetration rate of new molted 2nd‐instar *C. suppressalis* larvae within 90 min, (b) mass gain of larvae, and (c) Si content in the stem of rice plants. The controls were rice plants without sodium silicate amendment. For experiment (a) *n* = 3, for (b) *n* = 15–20, and for (c) *n* = 9. Values are mean ± *SEM*. Different letters indicate significant differences (ANOVA, *p* < .05, Fisher's test)

**FIGURE 2:**
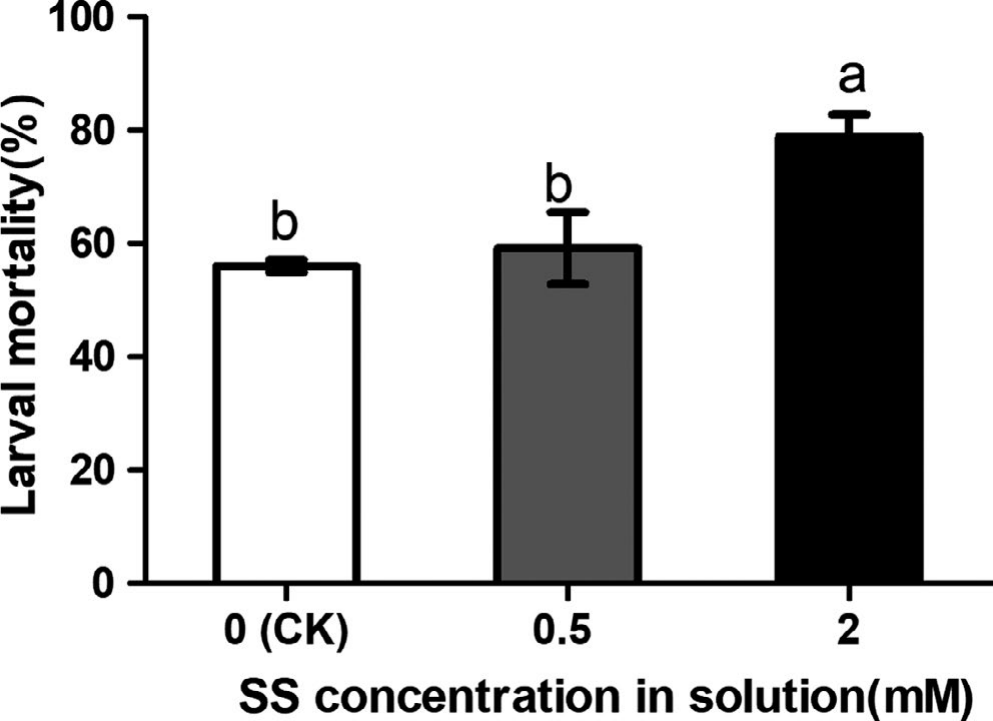
*Chilo suppressalis* larvae fed on rice plants treated with sodium silicate (SS) showed increased mortality to chlorpyrifos. Newly molted 2nd‐instar larvae were fed on the stems of rice plants for 3 days and then treated with chlorpyrifos. Mortality was recorded 24 hr after insecticide treatment. CK, control larvae were fed on rice plants without SS amendment. Values are mean ± *SEM* (*n* = 3). Different letters indicate significant differences (ANOVA, *p* < .05, Fisher's test)

To verify whether SS amendment enhances rice resistance to SSB larvae, rice plants were grown in a nutrient solution with SS amendment for 20 days were inoculated with SSB larvae. The percentage of penetrated larvae was significantly higher in control plants than that in Si‐treated rice plants (Figure 1a, *F*(2,6) *=* 5.432, *p* = .045). In addition, the weight of larvae fed on Si‐treated plants was significantly lower than those fed on the control plants (Figure 1b, *F*(2,52) *=* 13.72, *p* < .001). Moreover, SS amendment in nutrient solution significantly increased higher concentration of the silicon in the rice stems compared to rice plants without silicon amendment (Figure 1c, *F*(2,24) *=* 64.42, *p* < .001). Furthermore, sensitivities of SSB fed on Si‐treated rice to insecticide were evaluated. Larvae fed on 2 mM Si‐treated plants showed significantly higher mortality to chlorpyrifos compared to the larvae fed on rice plants without Si amendment (Figure 2, *F*(2,6) = 8.191, *p* = .0193). These results demonstrated that rice plants supplied with Si enhanced plant defense against SSB, by physically inhibiting penetration and increasing larval sensitivities to the chlorpyrifos insecticide.

### 3.2 Larval performance and insecticide sensitivity of SSB fed on artificial diet with sodium silicate amendment

**FIGURE 3:**
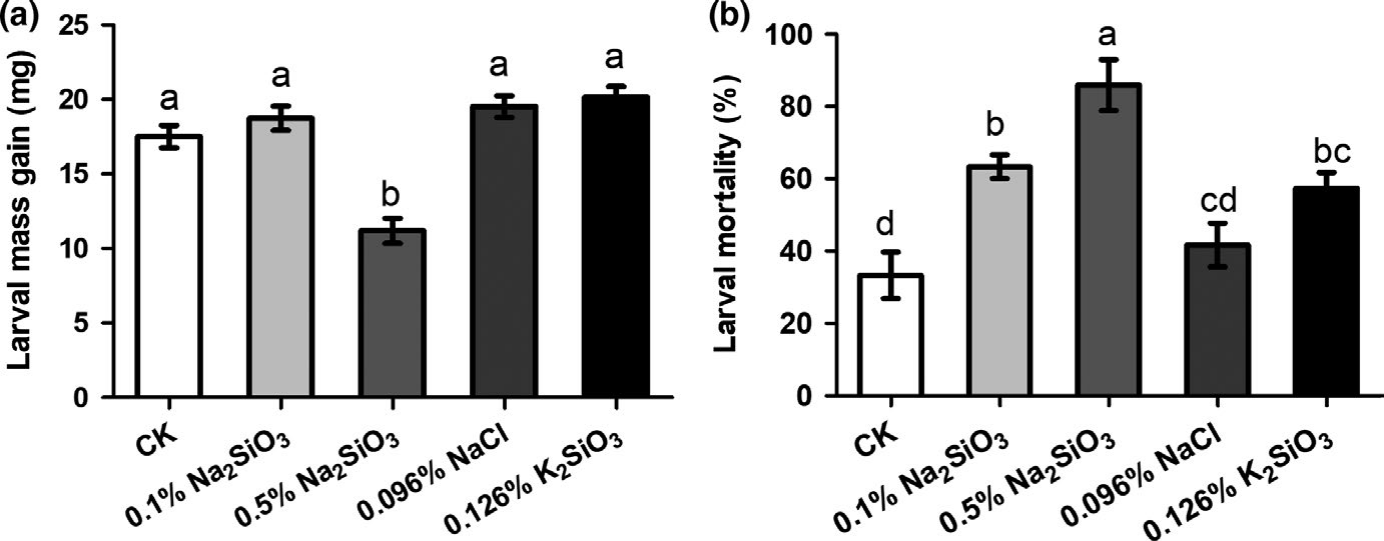
Silicon in the artificial diet suppressed the larval growth and enhanced the larval mortality to chlorpyrifos. (a) Newly molted 3rd‐instar larvae were fed on artificial diets with different chemical amendments for 2 days, and then, the mass was measured. (b) Mortality was recorded 24 hr after chlorpyrifos treatment. The controls were larvae fed on artificial diets without any amendments. Treatments of both 0.1% Na_2_SiO_3_ and 0.0096% NaCl had the same concentration of sodium in the artificial diet, and the treatment of 0.126% K_2_SiO_3_ had the similar concentration of silicon as that of 0.1% sodium silicate in the artificial diet. Values are mean ± *SEM*. For experiment (a) *n* = 20, and for (b) *n* = 3. Different letters indicate significant differences (ANOVA, *p* < .05, Fisher's test)

The mass of larvae fed on artificial diet supplemented with 0.096% sodium chloride, 0.1% sodium silicate, and 0.126% potassium silicate showed no difference to the controls, while the mass of larvae fed on artificial diet containing 0.5% sodium silicate significantly decreased compared to that of other four treatments (Figure 3a, *F*(4, 95) = 22.4, *p* < .05). Additionally, 0.096% sodium chloride had the same concentration of sodium as 0.1% sodium silicate, and 0.126% potassium silicate had the same concentration of silicic acid as 0.1% sodium silicate in the artificial diets. After treatment with chlorpyrifos, higher mortality of larvae fed on artificial diets containing 0.1% and 0.5% sodium silicate, as well as 0.126% potassium silicate, was observed compared to the control larvae fed on artificial diet containing 0.096% sodium chloride (Figure 3b, *F*(4, 10) = 13.29, *p* = .0005). These results indicated that it was silicon rather than sodium in the artificial diets that increase larval mortality of SSB to chlorpyrifos.

### 3.3 Insecticide sensitivity of SSB larvae fed on artificial diet containing the mixture of chlorpyrifos and sodium silicate

**FIGURE 4:**
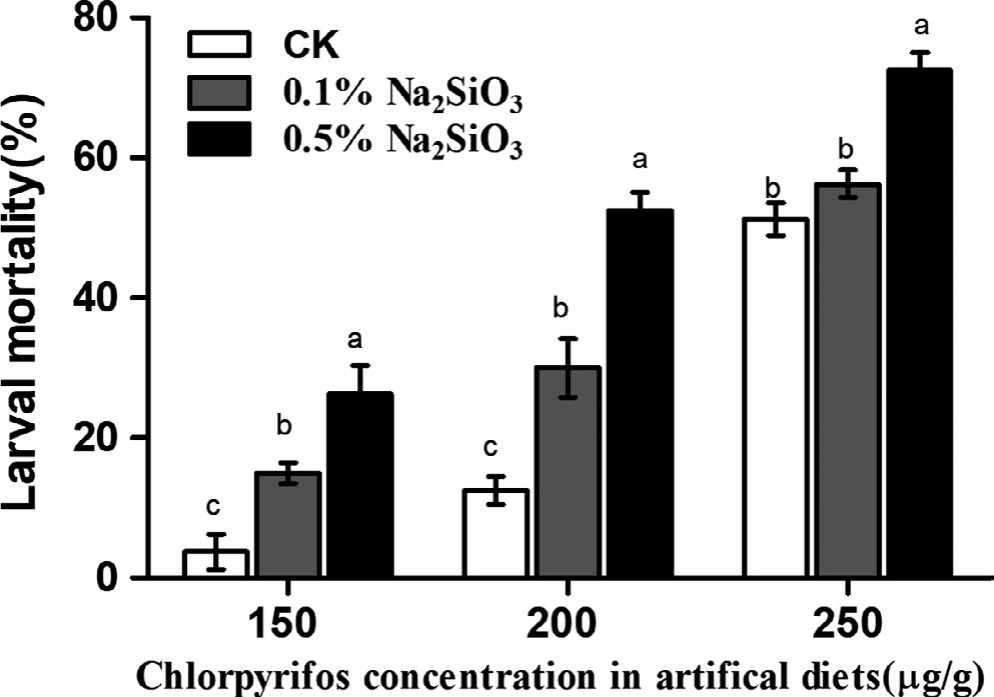
Silicon enhanced the toxicity of chlorpyrifos to *Chilo suppressalis* larvae. Newly molted 3rd‐instar larvae were fed on artificial diets containing the mixture of both sodium silicate and chlorpyrifos for 2 days, and then, the larval mortality was measured. Values are mean ± *SEM* (*n* = 5). Different letters indicate significant differences (ANOVA, *p* < .05, Fisher's test)

In order to investigate whether Si has synergistic effects on insecticide mortality, the mixture of chlorpyrifos and sodium silicate was applied to artificial diets to examine mortality of SSB larvae (Figure 4). The addition of 0.1% and 0.5% SS showed synergistic effects with chlorpyrifos by increasing the mortality of SSB larvae when the concentrations of chlorpyrifos reached 150 µg/g (*F*(2, 12) = 14.73, *p* = .0006) and 200 µg/g (*F*(2, 12) = 44.11, *p* < .001). Furthermore, the synergistic effect of 0.5% SS was significantly more effective than that of 0.1% SS, and when concentration of chlorpyrifos reached 250 µg/g, the synergistic effect of 0.5% SS was still very significant (*F*(2, 12) = 23.7, *p* < .001). The results showed that Si can enhance the toxicity of chlorpyrifos to SSB larvae.

### 3.4 Effects of sodium silicate amendment on detoxification enzymes in SSB larvae fed on rice plants and artificial diets

The activities of insect detoxification‐related enzymes were assayed in the midguts of SSB larvae fed on rice plants for 3 days and on artificial diets containing sodium silicate for 2 days (Table 2). The activities of AChE in SSB larvae fed on Si‐treated rice plants were significantly lower than that in SSB larvae fed on control rice plants without Si addition (*t*_(18)_ = 3.442, *p* = .0029). Similarly, the activities of both GST and CYP450 in the larvae fed on Si‐treated plants were significantly lower than that in larvae fed on control plants (GST, *t*_(18)_ = 5.811, *p* < .001; CYP450, *t*_(18)_ = 4.311, *p* = .0004). In artificial diet experiment, the activities of AChE in the larvae fed on Si‐treated diets were about fivefold lower than that of controls (*p* = .0261) (Table 2). Similarly, the activities of both GST and CYP450 in the larvae fed on Si‐treated artificial diet were significantly lower than that in control larvae (GST, *p* = .0434; CYP450, *p* = .0448). Overall data indicated that sodium silicate either applied to plants or artificial diets suppressed the activities of detoxification‐related enzymes in the midguts of SSB larvae.

**TABLE 2:** Activities of detoxification enzymes in the midguts of SSB larvae fed on rice plants or artificial diet supplied with sodium silicate (SS)

Treatments	Enzymes activities		
	AChE (U/mg∙prot)	GST (U/mg∙prot)	CYP450 (ng/ml)
Control plants	84.00 ± 2.810**	53.27 ± 1.206**	8.23 ± 0.130**
Plants with SS	72.96 ± 1.547	45.85 ± 0.418	7.49 ± 0.112
Control artificial diets	25.420 ± 5.842*	104.6 ± 13.860*	16.730 ± 0.164*
Artificial diets with SS	5.267 ± 0.188	63.880 ± 1.615	11.71 ± 1.733

Note

Control plants: SSB larvae fed on the stems of rice plants without additional sodium silicate; Plants with SS: SSB larvae fed on the stems of rice plants treated with 2 mM sodium silicate. Control artificial diets: SSB larvae fed on artificial diets without adding sodium silicate; Artificial diets with SS: SSB larvae fed on artificial diets applied with 0.1% sodium silicate (w/w). Values are mean ± *SEM* (*n* = 5). The asterisk indicates a significant difference between two treatments (*p* < .05 using unpaired *t* test).

### 3.5 Analysis of DEGs in the midgut of SSB larvae fed on Si‐amended artificial diets

**FIGURE 5:**
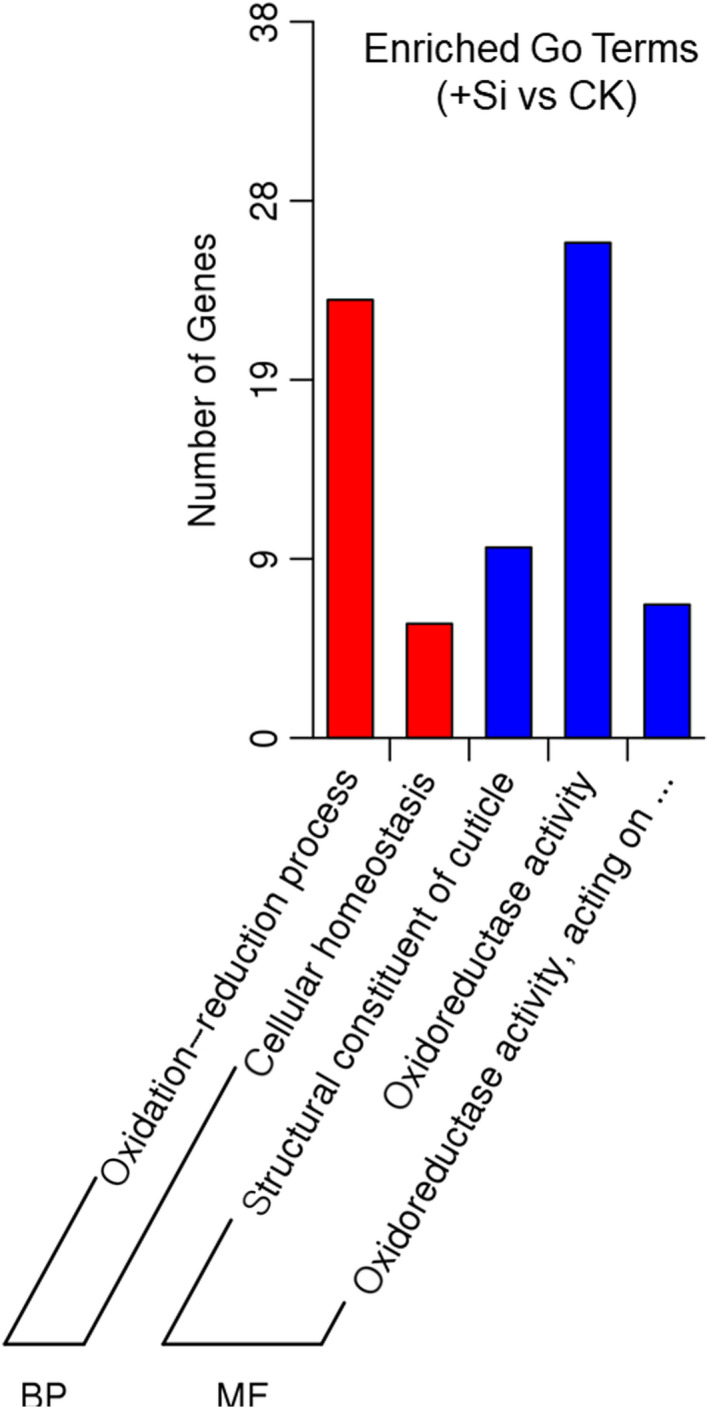
Gene ontology (GO) classifications of midgut genes of *Chilo suppressalis* larvae fed on artificial diets containing 0.1% sodium silicate (+Si) and larvae fed on normal artificial diets (CK). The different genes are assigned to two main categories: biological process (BP) and molecular function (MF). The y‐axis shows the number of genes matched in a category

To dissect the molecular mechanism underlying the SSB responses to sodium silicate treatment, we utilized RNA‐seq to explore differences in gene expression in midgut tissues of SSB larvae maintained on artificial diets with or without SS for 48 hr. Then, the midgut tissues were dissected and the DEGs were analyzed by RNA‐Seq. In total, 33 downregulated genes with transformation (log_2_ fold ≤ −2) were observed in the midgut of SSB larvae fed on artificial diets containing SS (Table 3). Most DEGs in SS‐treated caterpillars compared to control larvae were enriched in biological processes (oxidation–reduction and cellular processes) and molecular functions (structural constituent of cuticle and oxidoreductase activity) (Figure 5). These results showed that numerous putative metabolic enzymes especially those having oxidoreductase activity were suppressed in SSB subjected to SS treatment, which may help to understand why the detoxification‐related enzymes were mostly inhibited.

**TABLE 3:** Downregulated genes in SSB larvae fed on artificial diet with sodium silicate

	Gene_id	(+Si vs. CK) log2.Fold_change	NR Description
1	Cluster‐14641.26032	−2.0258	Hydroxyacylglutathione hydrolase
2	Cluster‐14641.25458	−2.1013	Stathmin isoform X2
3	Cluster‐14641.29694	−2.2709	Dihydrolipoamide dehydrogenase
4	Cluster‐14641.27867	−2.2969	Aldehyde dehydrogenase
5	Cluster‐14641.16417	−2.3537	Transketolase
6	Cluster‐14641.28810	−2.5602	Immulectin‐4
7	Cluster‐14641.28269	−2.604	Predicted protein
8	Cluster‐14641.37047	−2.6872	Larval cuticle protein LCP‐30 precursor
9	Cluster‐14641.35345	−2.6909	Attacin
10	Cluster‐12642.1	−2.6991	Probable chitinase 3
11	Cluster‐14641.4650	−2.756	Fatty acid synthase‐like
12	Cluster‐14641.39895	−2.7936	Cuticular protein RR‐1 motif 21 isoform X1
13	Cluster‐14641.15900	−2.8093	Cytochrome P450
14	Cluster‐14641.39252	−3.0014	Protease inhibitor‐like protein [Danaus plexippus]
15	Cluster‐14641.18081	−3.1222	Ubiquitin fusion degradation protein 1 homolog
16	Cluster‐14641.24460	−3.6619	Cuticular protein RR‐1 motif 42 precursor
17	Cluster‐14641.21747	−5.0779	Hypothetical protein KGM_21589
18	Cluster‐14641.21750	−5.1209	Hypothetical protein KGM_21589
19	Cluster‐14641.18724	−5.295	AMP deaminase 2 isoform X2
20	Cluster‐14641.14475	−5.3518	Uncharacterized protein LOC105398387 isoform X1
21	Cluster‐14641.19897	−5.3617	Hypothetical protein KGM_21589
22	Cluster‐420.0	−5.6793	Leech‐derived tryptase inhibitor C
23	Cluster‐14641.13991	−5.6907	Antennal esterase
24	Cluster‐14641.29718	−5.8908	Protein hu‐li tai shao
25	Cluster‐14641.30332	−5.9706	Juvenile hormone epoxide hydrolase‐like
26	Cluster‐2942.0	−5.996	Putative CCR4‐NOT transcription complex, subunit 1 isoform a
27	Cluster‐14641.26802	−6.1029	Putative nuclease HARBI1
28	Cluster‐14641.4910	−6.5712	Mucin‐5AC‐like
29	Cluster‐10311.0	−6.739	Uncharacterized protein LOC101746734
30	Cluster‐14641.31790	−6.9123	V‐type proton ATPase subunit D
31	Cluster‐14641.12090	−8.1027	Chitin deacetylase
32	Cluster‐14641.28282	−8.5331	B‐cell receptor‐associated protein 31
33	Cluster‐14641.39894	−8.8443	Uncharacterized protein LOC105078493

Note

The genes of with log2 transformation of fold changes lower than −2 were selected and displayed in the table.

### 3.6 Verification of RNA‐seq results by qRT‐PCR

**FIGURE 6:**
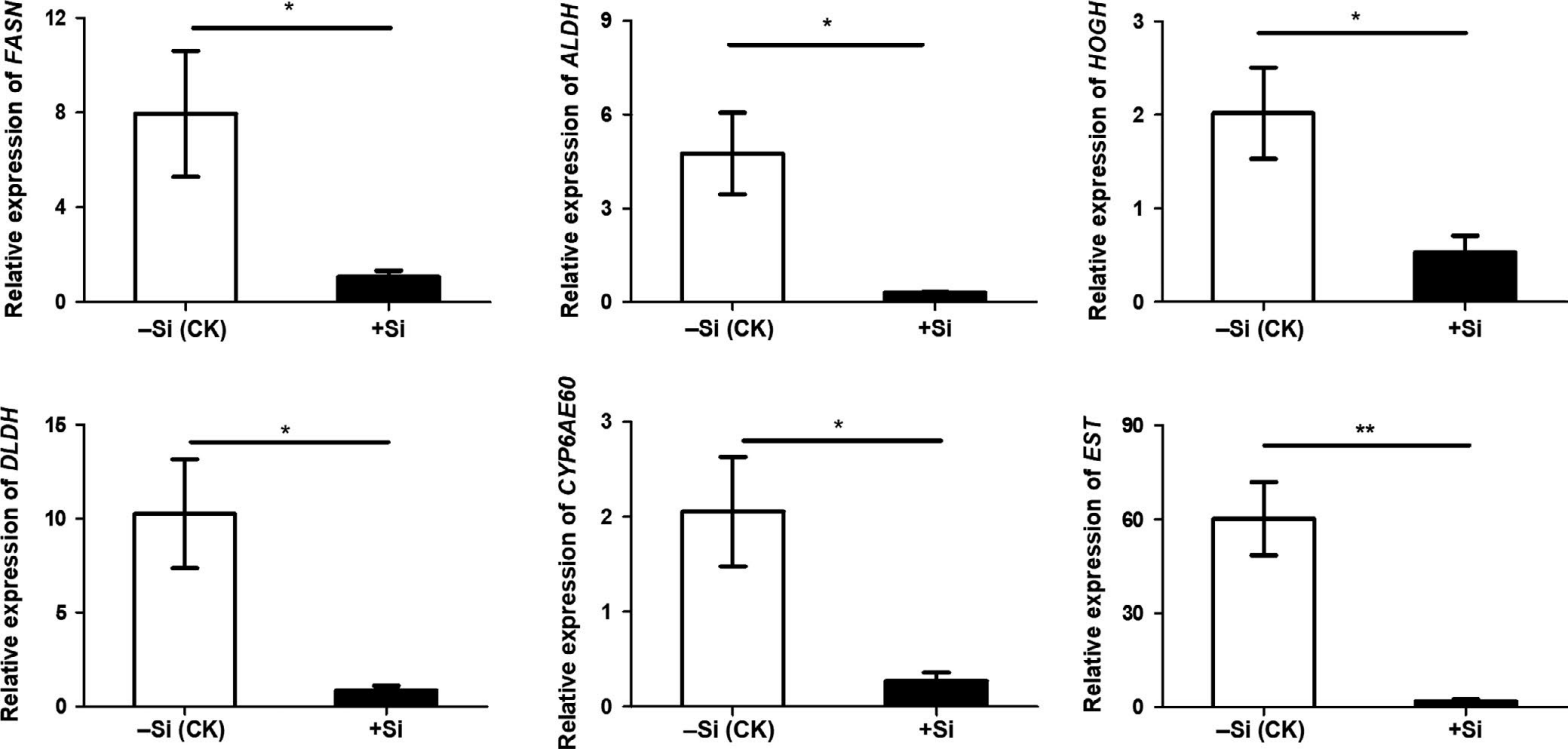
Silicon inhibited the expression of metabolism and detoxication‐related genes in the midguts of *Chilo suppressalis* larvae validated by qRT‐PCR. ‐Si (CK), larvae fed on normal artificial diets; +Si, larvae fed on artificial diets containing 0.1% sodium silicate (w/w). Midguts were collected from larvae that fed on artificial diets with/without silicon amendment for 48 hr. Values are mean ± *SEM* (*n* = 3–4). The asterisk indicates a significant difference between the two treatments (*p* < .05; unpaired *t* test). *p < .05; **p < .01

To validate the gene expression data obtained from RNA‐seq, we selected four genes which found to be downregulated in samples from sodium silicate treated larvae for qRT‐PCR analysis (Figure 6). *FASN* (fatty acid synthase) is a gene in fatty acid biosynthesis pathway, and *ALDH* (aldehyde dehydrogenase gene), *HOGH* (hydroxyacylglutathione hydrolase), and *DLDH* (dihydrolipoamide dehydrogenase) were genes in pyruvate metabolism pathway. In addition, we also found two significantly downregulated genes called *CYP6AE60* and *EST* (Esterase) in SS‐treated larvae. All of the downregulated genes from RNA‐seq data were also found downregulated in the qRT‐PCR analysis (*FASN*, 7.5‐fold; *ALDH*, 15.7‐fold; *HOGH*, 3.8‐fold; *DLDH*, 12‐fold; *CYP6AE60*, 7.6‐fold; and *EST*, 31.3‐fold, respectively). These results indicated that the RNA‐seq approach provided reliable DEG data for this assay and demonstrated that SS treatment has significantly adverse effects on these resistance‐related genes in the midguts of SSB larvae.

## 4 DISCUSSION

The most significant finding of this study is that larval feeding on either SS‐treated rice plants or SS‐contained artificial diet suppresses the activities of detoxification‐related enzymes and enhances the insecticide sensitivity of SSB larvae. Similarly, induction of plant resistance with exogenous application of methyl jasmonate to cotton plants (*Gossypium hirsutum* L.) reduced the activities of detoxification enzymes in the cotton pest *Helicoverpa armigera*; however, it was not clear which specific allelochemicals, if any, were detoxified (Yang, Wu, Xie, & Rantala, 2013).

Many studies have tried to elucidate the mechanisms for Si‐enhanced plant resistance to insect herbivores, including the direct defense through increased physical barrier (Han, Li, et al., 2016; Massey, Ennos, & Hartley, 2006) and indirectly through induction and priming of plant chemical defenses (GomesMoraes, Santos, & Goussain, 2005; Han, Li, et al., 2016; Ye et al., 2013). Si amendment improves rice Si content and impedes stalk penetration and prolongs penetration duration by *C. suppressalis* larvae, and larvae fed on the control plants without silicon treatment are bigger than those on Si‐treated rice plants (Hou & Han, 2010). Consistent with previous findings, here we found that the growth and penetration rate of SSB larvae on SS‐treated rice plants were significantly inhibited compared to control treatments (Figure 1a,b).

However, insoluble silicon may not influence the insect growth. The growth rates of the southern armyworm (*Spodoptera eridania*) at the highest level of insoluble silica treatment in artificial diets showed no difference to the control (Peterson & Coors, 1988). Similar study has found that addition of wollastonite at rates of up to 3.3% Si into artificial diet had no significant effect on larval weight of *Helicoverpa* spp. (Stanley, Baqir, & Mclaren, 2014). Thus, we suspected that soluble Si may be a key biochemical and physiological mediator. Therefore, in this study SSB larvae were reared on artificial diets containing a range of soluble Si levels, and Si significantly inhibited larval growth and tolerance to chlorpyrifos (Figure 3). It should be highlighted that the insect may be encountering many more different forms of silicon in the plant that in artificial diet, because sodium silicate absorbed by the rice plant occurred the conversion from silicic acid to silica. However, the major form of silicon in the xylem has been identified as monomeric silicic acid in rice, and the high concentration of silicic acid is transiently present in the xylem sap (Mitani, Ma, & Iwashita, 2005; Ma & Yamaji, 2006). Soluble silicic acid is formed by neutralization of SS with acid or hydrolyzed in solution (Danilovtseva, Aseyev, Karesoja, & Annenkov, 2009; Owusu, 1982), so SS could also be converted to silicic acid in artificial diet. Therefore, it may not be SS that is directly affecting the performance of SSB larvae, but the converted compound silicic acid. A previous study showed that exposure to silicic acid enhanced cytokinin biosynthesis and delayed senescence in both Arabidopsis and Sorghum (Markovich et al., 2017). In addition, silicic acid was able to inhibit the feeding of the brown planthopper on rice plants (Yoshihara et al., 1979). Further studies should pay more attention to the effects of silicic acid on both plants and herbivores.

According to the RNA‐seq results, SS was found to regulate the performance of SSB larvae through fatty acid biosynthesis and pyruvate metabolism pathways, and several genes related to these two pathways were suppressed in SSB larvae (Table 4). Chlorpyrifos is one of the most widely used organophosphorus insecticides (Lee, Strand, & Doty, 2012) and is widely used in paddy fields to control pests such as SSB and planthoppers (Kumar, Praveenkumar, Jeon, & Thajuddin, 2014). In addition to genes related to fatty acid biosynthesis and pyruvate metabolism pathways, many other genes, such as P450s, CarEs, and glutathione transferases, may also mediate Si‐triggered responses in SSB. Chlorpyrifos kills insects by competing with acetylcholine and inhibiting the activity of AChE, and organophosphorus resistance has been related to metabolic changes in target insects (Cheng et al., 2010; Fournier & Mutero, 1994; Jiang et al., 2009; Qu et al., 2003). According to our observation, another two significantly downregulated genes *EST* (Esterase) and *CYP6AE60* were found in SS‐treated larvae (Figure 6), and these two genes have been found to play a role in insecticide detoxification and xenobiotic metabolism (Ping, 2014; Wang et al., 2014). In insects, many pheromones and other chemical molecules are types of esters that could be hydrolyzed by esterases (Montella, Schama, & Valle, 2012). Therefore, further functional studies using targeted RNAi or gene editing approaches are needed to evaluate the impacts of silicate enzymes and genes in larval midguts.

**TABLE 4:** KEGG pathways containing genes expressed in SSB larvae with or without sodium silicate treatment

KEGG term	Unigenes	NR description
ko00061: Fatty acid biosynthesis	Cluster‐14641.4650	Fatty acid synthase
ko00620: Pyruvate metabolism	Cluster‐14641.27867 Cluster‐14641.26032 Cluster‐14641.29694	Aldehyde dehydrogenase Hydroxyacylglutathione hydrolase Dihydrolipoamide dehydrogenase

Insecticides are often used in combination with organosilicon adjuvants to facilitate the wetting and the spread of droplets on leaves, which result in a more uniform distribution of active ingredients (Srinivasan, Hoy, Singh, & Rogers, 2008; Stevens, Kimberley, Murphy, & Policello, 1993), and organosilicon adjuvants may also show insecticidal activity to several pests (Cocco & Hoy, 2014). Organosilicon added to chlorpyrifos showed synergistic activity to increase the mortality of *C. suppressalis* larvae in laboratory by spraying method (Zhang, Chen, Lu, Bao, & Deng, 2009). However, no information is available about whether inorganic silicon such as sodium silicate could be used as an adjuvant to insecticides. In current study, larvae fed on artificial diets containing Si and chlorpyrifos showed higher mortality compared to larvae fed on diets containing only chlorpyrifos (Figure 4). Sodium silicate was observed to have synergistic activity to chlorpyrifos, suggesting that application of sodium silicate to either plants or insects has a great potential to effectively control pests using a lower amount of chemical insecticides.

Si has been considered a plant resistance elicitor to control pests (Debona, Rodrigues, & Datnoff, 2017; Keeping & Kvedaras, 2008; Liang et al., 2015; Reynolds, Keeping, & Meyer, 2009; Sakr, 2017; Ye et al., 2013). Current study indicates that sodium silicate has direct effects on insect performance by interfering larval growth and inhibiting activities of detoxification related with enzymes. Additionally, sodium silicate showed a synergistic activity to chlorpyrifos and accelerates mortality of SSB larvae. In conclusion, sodium silicate not only can be used as a resistance inducer for plants, but also can be used as a potential and effective addition to integrated pest management tactics. This expands an interesting and unique dimension to our current understanding of how Si may mediate plant–insect interactions.

## CONFLICT OF INTEREST

None declared.

## AUTHOR CONTRIBUTION

**Jie Wang:** Funding acquisition (lead); methodology (equal); project administration (lead); writing – review and editing (equal). **Rongrong Xue:** Data curation (lead); writing – original draft (equal); writing – review and editing (equal). **Hui Yan:** Data curation (equal). **Xueyang Ju:** Data curation (equal). **Zhou Gao:** Data curation (equal); investigation (equal). **Lin Hu:** Methodology (equal); writing – review and editing (equal). **Mohammed Esmail Abdalla Elzaki:** Methodology (equal); writing – review and editing (equal). **Rensen Zeng:** Writing‐review and editing (equal). **Yuanyuan Song:** Funding acquisition (equal); resources (equal); writing – review and editing (equal).

## Data Availability

The raw sequence reads obtained from RNA‐seq were submitted to NCBI Sequence Read Archive (SRA) under BioProject PRJNA554390. All data for generation of figures in this publication are deposited in the Dryad (https://doi.org/10.5061/dryad.2bvq83bm9).
